# Assessment of long non-coding RNA expression reveals novel mediators of the lung tumour immune response

**DOI:** 10.1038/s41598-020-73787-6

**Published:** 2020-10-09

**Authors:** Adam P. Sage, Kevin W. Ng, Erin A. Marshall, Greg L. Stewart, Brenda C. Minatel, Katey S. S. Enfield, Spencer D. Martin, Carolyn J. Brown, Ninan Abraham, Wan L. Lam

**Affiliations:** 1 Department of Integrative Oncology, British Columbia Cancer Research Centre, 675 West 10th Avenue, Vancouver, BC V5Z 1L3 Canada; 2 Department of Medical Genetics, University of British Columbia, Vancouver, BC Canada; 3 Department of Zoology, University of British Columbia, Vancouver, BC Canada; 4 Department of Microbiology and Immunology, University of British Columbia, Vancouver, BC Canada

**Keywords:** Cancer, Computational biology and bioinformatics, Immunology

## Abstract

The tumour immune microenvironment is a crucial mediator of lung tumourigenesis, and characterizing the immune landscape of patient tumours may guide immunotherapy treatment regimens and uncover novel intervention points. We sought to identify the landscape of tumour-infiltrating immune cells in the context of long non-coding RNA (lncRNAs), known regulators of gene expression. We examined the lncRNA profiles of lung adenocarcinoma (LUAD) tumours by interrogating RNA sequencing data from microdissected and non-microdissected samples (BCCRC and TCGA). Subsequently, analysis of single-cell RNA sequencing data from lung tumours and flow-sorted healthy peripheral blood mononuclear cells identified lncRNAs in immune cells, highlighting their biological and prognostic relevance. We discovered lncRNA expression patterns indicative of regulatory relationships with immune-related protein-coding genes, including the relationship between *AC008750.1* and *NKG7* in NK cells. Activation of NK cells in vitro was sufficient to induce *AC008750.1* expression. Finally, siRNA-mediated knockdown of *AC008750.1* significantly impaired both the expression of *NKG7* and the anti-tumour capacity of NK cells. We present an atlas of cancer-cell extrinsic immune cell-expressed lncRNAs, in vitro evidence for a functional role of lncRNAs in anti-tumour immune activity, which upon further exploration may reveal novel clinical utility as markers of immune infiltration.

## Introduction

The remarkable success of immunotherapy in non-small cell lung cancer (NSCLC) has highlighted the contribution of infiltrating immune cells to tumour control. In particular, uncovering the mechanisms used by lung tumours to evade natural anti-tumour immunity has resulted in the development of therapeutic agents that revitalise the immune cell response against tumours by direct targeting of inhibitory receptors^1,2^. Despite these successes, a key outstanding clinical challenge is the wide variation in patient response, with treatments only effective in 36–48% of patients with advanced metastatic NSCLC^3–5^. Numerous tumour-intrinsic and extrinsic features have demonstrated prognostic value, including mutational burden, mismatch repair deficiencies, CD8 + T cell infiltration, and expression of interferon-gamma gene signatures^6–8^. For example, a higher proportion of cytotoxic CD8 + T cells infiltrating the tumour is associated with improved outcome for NSCLC patients^9^. With growing availability of sequence data derived from bulk patient tumour samples, the capability of accurate immunophenotyping from omics data (based on cell type-specific gene expression) would enable the exploration of new molecular immunologic features of the tumour microenvironment in the search for disease and treatment markers^10^.

Demarcating immune cell infiltrate and predicting response to immunotherapy through high-throughput sequencing of tumour samples would augment traditional immunohistochemistry approaches. For example, sequencing data provides information on multiple tumour molecular features, such as mutational load, genetic abnormalities, and notably, non-coding RNAs, which are likely to play an important role in the tumour immune microenvironment^11^. Expressed non-coding transcripts exceeding 200 nucleotides are classified as long non-coding RNAs (lncRNAs), which modulate gene expression. LncRNA transcripts can regulate other genes in *cis* or in *trans* through a multitude of mechanisms, such as transcriptional enhancers, decoys for microRNAs, and molecular scaffolds for protein and/or other RNA transcripts^12^. Early studies on lncRNAs focused on their deregulation in cancer, but many have since revealed far more extensive gene-regulatory roles in development and disease^13–15^.

LncRNAs have been shown to be key regulators of immune cell development and plasticity^16^. Indeed, lncRNAs have been shown to act through mechanisms that encompass the full range of lncRNA-mediated gene regulation, including nucleic acid scaffolding during V(D)J recombination^17^, differentiation of dendritic cells via transcription factor binding^18^, chromatin modification resulting in negative regulation of *FOXP3*^19^, and ribonuclear complex organisation during cytosolic DNA sensing^20^. However, the relative contribution of tumour and immune cell-derived lncRNAs in bulk tumour sequencing data and their relevance to natural or therapeutic anti-tumour immunity is only beginning to be appreciated.

Recent in vitro work has shown that specific lncRNAs identified in a variety of immune cell lines have the potential to be prognostic^21^. This is particularly applicable to immunotherapy, in which gene expression profiling approaches have been used to explore mRNA expression of immune-related genes that may be indicative of distinct immune cell types within tumours^22^. Indeed, immune-gene expression such as *NKG7*, *IDO1*, and *IFNG*, represent an active immune response, and are differentially expressed in responsive and non-responsive patients treated with immunotherapy^23,24^. Further, a recent study found that the expression of lncRNAs with putative immune-related function can be used to guide the molecular and immunological sub-classification of tumours, which can be used to supplement treatment decisions and prognosis^25^.

Here, we assess the roles of lncRNAs in the anti-tumor immune response. We analyzed expression of lncRNAs from two cohorts of NSCLC tumours and matched non-malignant tissue and identified lncRNAs deregulated in tumour samples, observations which we illustrate may result from a higher proportion of tumour-infiltrating immune cells. Separate cohorts of single cell RNA sequencing data from tumour-infiltrating healthy cells and bulk sequencing of sorted peripheral blood cells from healthy donors revealed immune-subtype specific expression of a large number of lncRNAs, including some that were seen in the tumour-associated list. Finally, using siRNA knockdown of a specific lncRNA (*AC008750.1*), we found that immune-mediated killing of tumour cells was inhibited, identifying a possible role for lncRNA transcripts in anti-tumour immunity.

## Methods

### Tissue samples

Thirty-six pairs of fresh frozen lung adenocarcinoma (LUAD) tumours and adjacent non-malignant tissue were obtained from the Tumour Tissue Repository of the British Columbia Cancer Agency (BCCA) under written informed consent. Samples were obtained under relevant ethical regulatory protocols, which were approved by the University of British Columbia-BCCA Research Ethics Board (Certificate number H15-03060, Table 1). All research was performed in accordance with relevant regulations. Total RNA was extracted using Trizol reagent (ThermoFisher, MA) from microdissected histological sections with > 80% tumour cell content as reviewed by a lung pathologist as previously described in Ref.^26^. RNA sequencing was performed using the Illumina HiSeq platform as previously described in Ref.^27^.

**Table 1:** Sample cohort characteristics.

Samples	TCGA-LUAD	BCCA-LUAD	E-MTAB-6149	GSE60424^a^
	*Malignant*—54 *Non-malignant*—54	*Malignant*—36 *Non-malignant*—36	*Malignant*—15 (3 samples/patient)	*Sorted blood*—4^b^
Tumour cell content	> 60%	> 90%	N/A	N/A
**Clinical information**				
Mean age	67	70	70	28
**Gender**				
Male	24	10	3	–
Female	30	26	2	4
**Ethnicity**				
Caucasian	51	11	–	3
Asian	–	14	–	–
Hispanic/Latino	–	–	–	1
Other	3	11	–	–
Not reported	–	–	5	–
**Stage**				
I	28	20	2	–
II	14	11	2	–
III	10	3	1	–
IV	2	1	–	–
**Smoking**				
Current	7	5	1	–
Former	36	6	4	4
Never	5	25	–	

^a^*Avg Cell Count (millions)*: B—1.01; CD4 T—1.07; CD8 T—1.01; Monocytes—2.00; Neutrophils—14.7; NK—0.379.

^b^GSE60424 also contains data from 4 disease states (Amyotrophic Lateral Sclerosis, Multiple Sclerosis, Sepsis, Type 1 Diabetes), although the healthy donor samples were what was used for analysis of lncRNA expression.

**Figure 1:**
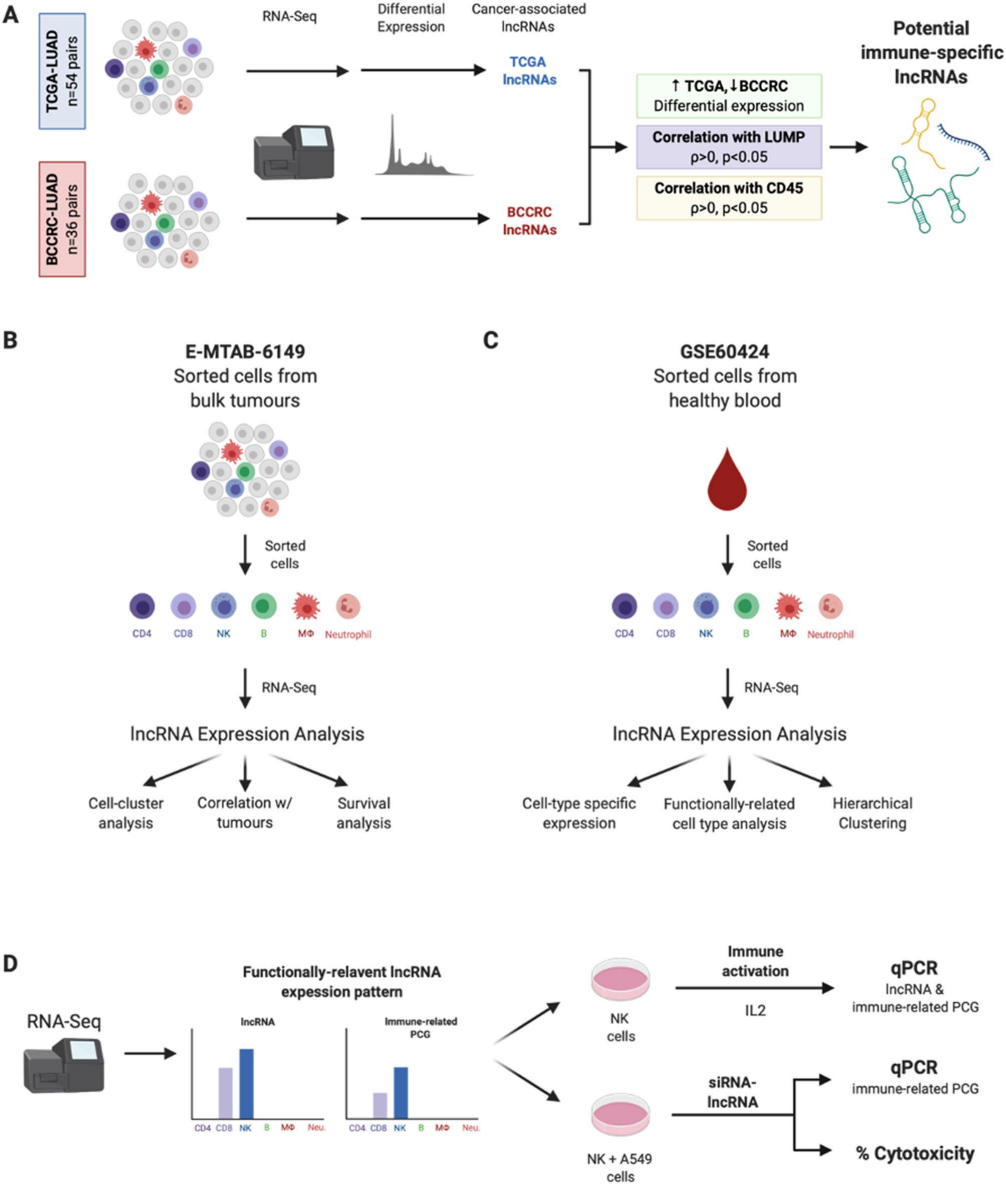
Overall analysis process. (**A**) Identification of potential cancer-cell-extrinsic lncRNAs through analysis of bulk RNA-sequencing data from the BCCRC- and TCGA-LUAD cohorts. (**B**) Exploration of lncRNA expression in single-cell RNA sequencing data of immune cells isolated from lung tumour samples (E-MTAB-6149). (**C**) Analysis of lncRNA expression in healthy human immune cells (GSE60424). (**D**) Assessment of lncRNA-based anti-tumour activity in vitro through both immune activation and siRNA-based assays.

Paired LUAD and non-malignant (n = 54 pairs, Table 1) RNA sequencing data from The Cancer Genome Atlas (TCGA) were downloaded from CancerBrowser (Illumina HiSeq, https://genome-cancer.ucsc.edu/proj/site/hgHeatmap/). Single cell RNAseq data of five NSCLC tumours were obtained from E-MTAB-6149 (Table 1)^28^. RNA sequencing data of FACS-sorted immune cells (CD8 + T, CD4 + T, B, monocyte, neutrophils, and NK) from a cohort which included samples healthy donors were obtained from GSE60424 (Table 1)^29,30^. The overall analysis process is illustrated in Fig. 1.

Raw sequencing reads were aligned to the hg38 build of the human genome using STAR 2.4.1d, and aligned reads were quantified against the Ensembl v89 reference gene annotations using Cufflinks v2.2.1^30,31^. Annotated gene types included for further analysis were antisense, bidirectional promoter lncRNA, lincRNA, and macro lncRNA for a total of 13,006 genes. When analyzing each cohort for lncRNA expression, we only considered only genes with summed FPKM > 1 across the cohort.

### Differential expression analysis

Differentially expressed lncRNAs were identified using a two-tailed Mann–Whitney U test and filtered by p < 0.05 following correction for multiple testing using the Benjamini–Hochberg method. Unsupervised hierarchical clustering was performed using a cluster distance metric of average linkage and the point distance metric as Pearson correlation. All analyses were made using MATLAB R2013a, RStudio v3.3.3, or Partek Flow build 7.0.18.0724.

### Survival analysis

To test if expression of a lncRNA correlated with patient survival, we identified patients in the top and bottom tertile of expression (“high” vs. “low” expression). Patients were categorised by vital status and days to death/last follow-up annotation. To determine if survival observations could be driven by other clinical cofactors, we used a multivariate model (Cox Proportional-Hazards) to account for age, sex, stage, and smoking history in the TCGA cohort (Supplemental Figure S1). To compare curves between high and low expression tertiles, the log-rank test was used. All analyses were made using GraphPad Prism 8 (GraphPad Software).

### *Cell lines and *in vitro* cytotoxicity assays*

NK92 and A549 cells were obtained from ATCC, fingerprinted, and verified as mycoplasma-free. Cells were maintained in RPMI (Thermo Fisher) supplemented with 10% fetal bovine serum (Thermo Fisher), L-glutamine (2 mmol/L, Thermo Fisher), penicillin (100 U/mL, Thermo Fisher), and streptomycin (0.1 mg/mL, Thermo Fisher).

NK92 cells were stimulated with PMA (100 ng/mL; Sigma) and ionomycin (0.5 uM; Sigma), anti-NKG2D, or IL-2 (50U/mL; Peprotech) and IL-15 (10 ng/mL; Peprotech) for 4 h. Total RNA from cell lines was isolated using the QIAcube (Qiagen), and cDNA synthesis was carried out with the High Capacity Reverse Transcription Kit (Applied Biosystems) with an added RNase inhibitor (Promega). Purified cDNA was used to quantify *AC008750.1*, *NKG7*, and *HPRT* using the following primer sequences:

*AC008750.1* | F: GCCACGCCTCCTCTTAACC R: GGGACTCATCTCAACGGCAT.

*NKG7* | F: GCACCGATTTCTGGTTTGAGGC R: CAGCCATAATGCTGAAGGTCTGC.

*HPRT* | F: TGACACTGGCAAAACAATGCA; R: GGTCCTTTTCACCAGCAAGCT.

Values were normalised to HPRT expression using the ΔΔC_T_ method.

NK92 cells were transfected with custom siRNAs to *AC008750.1* or a scramble siRNA as a control (Thermo Fisher) and co-cultured with A549 cells at varying effector:target ratios. Cytotoxicity was assessed after 4 h of co-culture using the LDH release assay kit according to manufacturer’s instructions (Abcam).

### Statistical analysis

Statistical comparisons were made using GraphPad Prism 8 (GraphPad Software). Parametric comparisons of normally distributed values that satisfied the variance criteria were made by unpaired Student’s t-tests or One-Way Analysis of Variance (ANOVA) tests. Data that did not pass the variance test were compared with non-parametric two-tailed Mann–Whitney Rank Sum tests or ANOVA on Ranks tests.

### Ethics approval and consent to participate

Samples from the BC Cancer Research Centre (BCCRC) were obtained under written informed consent approved by UBC and BC Cancer Research Ethics Boards.

## Results

### Bulk tumour RNA sequencing data contains cancer-cell-extrinsic lncRNAs

A growing number of long non-coding RNAs have been functionally implicated in tumour development, growth, and metastasis^14,27,32^. Notably, lncRNA expression patterns display a high degree of cancer subtype specificity, likely reflecting tissue- and context-specificity^32^. Thus, we performed differential expression analysis in two cohorts of matched tumour and non-malignant transcriptomes (BCCA-LUAD, TCGA-LUAD). Both cohorts comprised primarily early-stage tumours (Stage I and II: 86% BCCA-LUAD, 78% TCGA-LUAD) and were predominantly female (72% BCCA-LUAD, 56% TCGA-LUAD). However, the two cohorts differed by smoking status (Current or former smoker: 31% BCCA-LUAD, 80% TCGA-LUAD) and ethnicity (Caucasian: 28% BCCA-LUAD, 94% TCGA-LUAD; Asian: 72% BCCA-LUAD, 0% TCGA-LUAD). The median tumour cell content as assessed by histology also differed between the two cohorts (> 90% BCCA-LUAD, > 60% TCGA-LUAD). First, we found 679 lncRNAs to be differentially expressed (corrected p < 0.05) between malignant and non-malignant samples in the TCGA cohort (Supplemental Table S1), and 752 in the BCCRC cohort (Supplemental Table S2), suggestive of a potential cancer-associated role for these deregulated lncRNAs. However, the BCCRC-LUAD cohort consists of samples microdissected to > 80% tumour cell content, while the TCGA-LUAD cohort has varying tumour purity ranging from 15 to 90%^33^.

We reasoned that some of these differentially expressed lncRNAs may simply be indicative of different cell types present in the sample; thus, we considered whether the cellularity of the tumour samples might affect the observed deregulated expression of these lncRNAs. To this end, we found that of the 385 lncRNAs found to be differentially expressed in both cohorts, only 273 displayed concordant deregulation patterns between tumour and matched non-malignant samples in their respective cohort (Table 2; Supplemental Table S3).

**Table 2:** Top 10 concordantly deregulated lncRNAs in TCGA-LUAD and BCCA-LUAD.

lncRNA	Fold change (BCCA)	Fold change (TCGA)	Direction of differential expression
*AC007128.1*	3.13236E+39	3.13638E+31	Up tumours
*UCA1*	3.29592E+37	35.90110987	Up tumours
*DNAJC27-AS1*	7.39619E+36	0.951087237	Down tumours
*AC079779.4*	7.18391E+31	0.210852767	Down tumours
*LINC01016*	2.88541E+30	0.538567561	Down tumours
*WDR11-AS1*	4.99702E+29	0.253491849	Down tumours
*FEZF1-AS1*	3.08818E+29	2,833,152.032	Up tumours
*ALKBH3-AS1*	1.86986E+29	0.933692846	Down tumours
*ALDH1L1-AS2*	8.90476E+27	0.596765073	Down tumours
*LINC00896*	2.52614E+26	5.531802561	Up tumours

Strikingly, the vast majority of the 112 lncRNAs that were discordantly deregulated displayed decreased expression in tumour samples of the microdissected BCCRC cohort but increased expression in tumours of the TCGA cohort, suggesting that these might be derived from non-cancer cells in the sample (Fig. 1). As lung adenocarcinomas are known to be highly infiltrated with immune cells, we looked for putative immune cell-derived lncRNAs that positively correlated with expression of *PTPRC*, encoding CD45 as a marker for leukocytes (Table 3; Supplemental Table S4). As an orthogonal approach, we correlated the expression of lncRNAs with tumour cellularity (Leukocytes Unmethylation for Purity (LUMP) scores), an established marker of immune cell infiltrate in tumours which assesses methylation at sites frequently methylated in tumour cells but not in immune cells (Table 4; Supplemental Table S5)^33^. Together, these results highlight that a proportion of lncRNAs observed to be deregulated in bulk tumour sequencing data may be the result of the cellularity of the samples, particularly infiltrating immune cells in immunogenic tumour types.

**Table 3:** Top 10 lncRNAs positively correlated with *PTPRC* in TCGA-LUAD.

lncRNA	Spearman R	95% confidence interval	P-value (two-tailed)
*LINC00861*	0.7317	0.6851–0.7723	< 0.0001
*LINC00426*	0.7252	0.6777–0.7667	< 0.0001
*LINC00528*	0.5903	0.5259–0.6480	< 0.0001
*CHRM3-AS2*	0.5856	0.5206–0.6438	< 0.0001
*C9orf139*	0.5536	0.4853–0.6152	< 0.0001
*LINC00494*	0.5513	0.4828–0.6132	< 0.0001
*LINC00877*	0.5472	0.4782–0.6094	< 0.0001
*LINC00158*	0.5052	0.4322–0.5717	< 0.0001
*LINC00539*	0.4791	0.4037–0.5480	< 0.0001
*IL21R-AS1*	0.4571	0.3800–0.5280	< 0.0001

**Table 4:** Top 10 lncRNAs negatively correlated with LUMP score in TCGA-LUAD.

lncRNA	Spearman R	95% confidence interval	P-value (two-tailed)
*C9orf139*	− 0.4383	− 0.5159 to − 0.3535	< 0.0001
*IL21R-AS1*	− 0.4637	− 0.5390 to − 0.3812	< 0.0001
*LINC00494*	− 0.4714	− 0.5459 to − 0.3895	< 0.0001
*LINC00525*	− 0.4728	− 0.5471 to − 0.3910	< 0.0001
*LINC00158*	− 0.4749	− 0.5490 to − 0.3933	< 0.0001
*LINC00582*	− 0.4789	− 0.5527 to − 0.3978	< 0.0001
*CHRM3-AS2*	− 0.5377	− 0.6053 to − 0.4623	< 0.0001
*LINC00861*	− 0.5596	− 0.6248 to − 0.4866	< 0.0001
*LINC00426*	− 0.6144	− 0.6733 to − 0.5479	< 0.0001
*LINC00528*	− 0.6286	− 0.6857 to − 0.5638	< 0.0001

### LncRNA expression from immune cells of the lung tumour microenvironment

**Figure 2:**
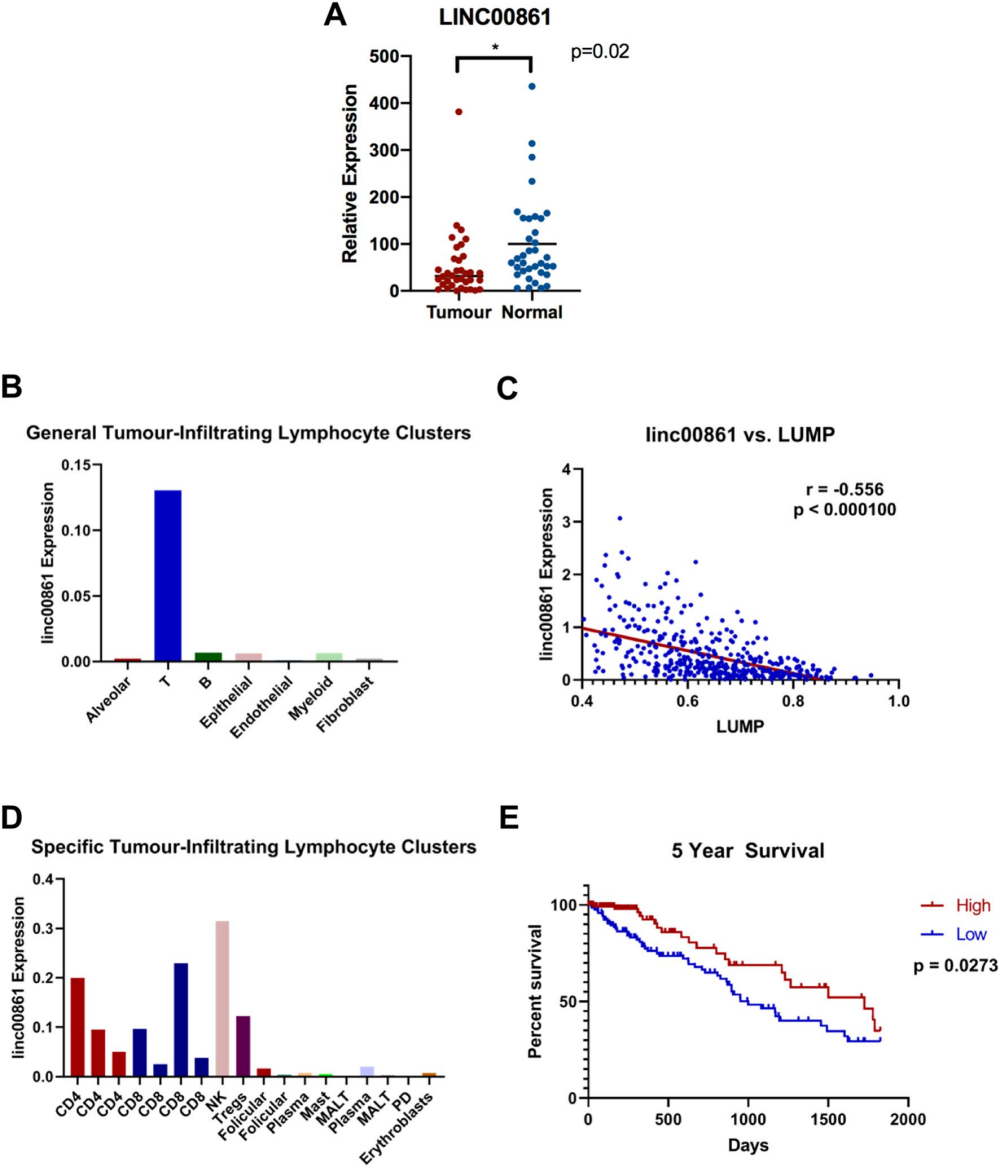
LncRNAs are widely expressed in the lung tumour microenvironment. (**A**) *linc00861* is underexpressed in microdissected tumour samples from BCCA-LUAD (**B**) *linc00861* displays preferential expression in various T cell subsets in tumour-derived immune cells of the E-MTAB-6149 scRNAseq dataset. (**C**) Expression of *linc00861* is significantly negatively correlated with the LUMP marker of immune infiltrate in the TCGA-LUAD cohort. (**D**) *linc00861* expression in E-MTAB-6149 is heavily skewed towards T cells in clusters of stromal cells isolated as single cells from bulk tumour samples. (**E**) Kaplan–Meier analysis of five-year overall survival in all tumour samples of the TCGA-LUAD cohort (n = 502). Patients within lower tertile of *linc00861* expression display significantly poorer 5-year overall survival.

As our initial analysis in cohorts of bulk tumour RNA sequencing data found that cellularity can affect lncRNA expression in tumour samples, we examined lncRNA expression in a third cohort (E-MTAB-6149) of single cell RNA sequencing (scRNAseq) of five NSCLC samples^28^. The original analysis of these data yielded 52 non-malignant stromal cell subsets, including 9 subsets each of T and B cells, in which we assessed lncRNA expression. As a representative example, we identified *linc00861*, which was our top hit in our analysis of lncRNAs positively correlated with CD45 (Table 3) was significantly underexpressed in microdissected BCCA tumour samples (Fig. 2A). *linc00861* was expressed predominantly in immune cells in scRNAseq data (Fig. 2B, Supplemental Table S6) and negatively correlated with LUMP score in TCGA-LUAD (Fig. 2C).

Mapping of *linc00861* to individual immune cell clusters based on marker gene expression used in the initial analysis of E-MTAB-6149 revealed highest expression in NK cells, with intermediate expression in CD4 + and CD8 + clusters (Fig. 2D); expression was also observed in the cluster attributed to regulatory T cells. Interestingly, T cells were a major source of lncRNA expression in the lung tumour microenvironment, with 111 lncRNAs displaying at least 1.5-fold greater expression in T cells relative to all other non-tumour cell types analyzed.

Given that non-coding RNAs represent a promising class of biomarkers, we explored the utility of *linc00861* to predict patient outcome. When stratified by *linc00861* expression, patients in the TCGA cohort with high expression of this immune-related lncRNA are significantly associated with better prognosis, which aligns with similar observations for patients with elevated levels of cytotoxic T cells (Fig. 2E). Notably, the biggest difference in slope was observed between linc00861-high and -low patients, potentially reflecting a role of cytotoxic immune cells in early tumour control. Multivariate Cox Regression analysis showed that this stratification in outcome was independent of age, sex, or smoking status, with stage being the only clinical variable significantly associating with survival (sex: p = 0.9809; age at diagnosis: p = 0.1366; smoking history: p = 0.0526; stage: p = 0.0000107; Fig. S1). Thus, lncRNA expression from immune cells in lung tumours may be used to inform both patient outcome and tumour immunology.

### LncRNAs are expressed in healthy immune cells

**Figure 3:**
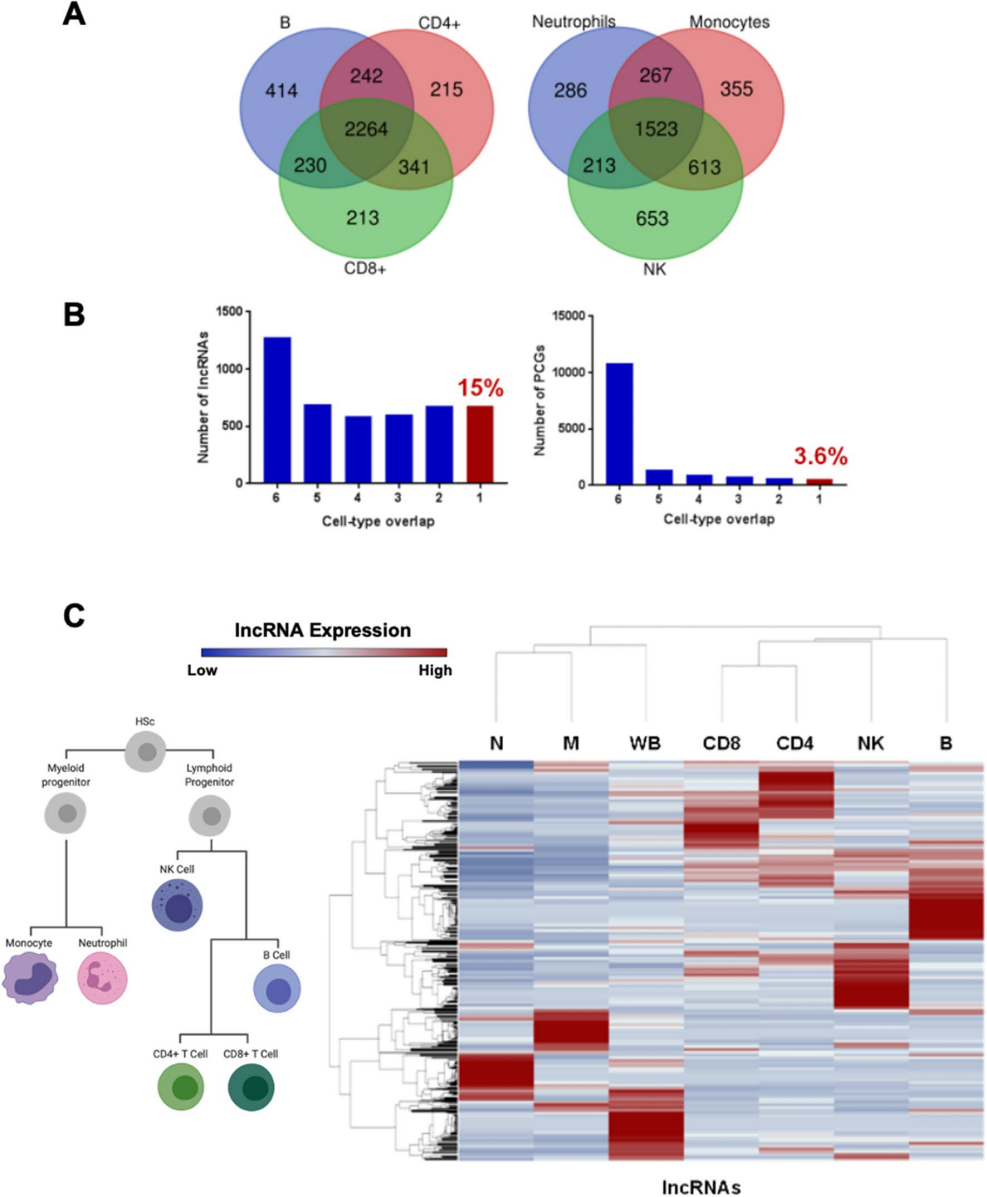
LncRNA expression in purified healthy human immune cells reveals cell-type specificity. (**A**) LncRNAs are expressed in adaptive (left) and innate (right) healthy human immune cells. (**B**) A greater proportion of lncRNAs display expression in one specific immune cell subset (red bar, left histogram) as compared to their protein-coding gene (PCG) counterparts (red bar, right histogram). (**C**) The expression of lncRNAs (blue: relatively low expression, red: relatively high expression) is able to recapitulate immune cell differentiation patterns (depicted on the left) in unsupervised hierarchical clustering analysis. and further highlights their cell-type-specific expression patterns. *HSC* hematopoietic stem cell, *N* neutrophils, *M* monocytes, *WB* whole blood, *CD8* CD8^+^ T cells, *CD4* CD4^+^ T cells, *NK* natural killer cells, *B* B cells.

As we observed lncRNA expression from tumour-infiltrating lymphocytes, we sought to further characterize lncRNA expression in a cohort of sorted human immune cells (GSE60424) to investigate their possible roles in normal immune function. RNA sequencing data from sorted CD8 + T cells, CD4 + T cells, B cells, NK cells, monocytes, neutrophils, and whole blood from four healthy donors along with the other disease state samples of the GSE60424 dataset were processed as above for lncRNA expression. We detected 4919 expressed lncRNAs (summed FPKM > 1 across all samples, subsequent summed FPKM > 0) in this cohort of immune cells, out of 13,006 genes annotated in Ensembl v89 (Supplemental Table S7). We found that approximately 25% of these expressed lncRNAs were expressed in all immune cell types analysed in this cohort, potentially suggesting a conserved biological function of these transcripts (Fig. 3A, Supplemental Table S8). In contrast, 15% of lncRNAs were exclusively expressed in one cell type as compared to 3% of protein-coding genes, consistent with described tissue- and context-specific expression patterns of lncRNAs (Fig. 3B, Supplemental Table S9). Notably, unsupervised hierarchical clustering of cell types based on lncRNA expression pattern largely recapitulated haematopoietic lineage development (Fig. 3C). Altogether, our analysis highlights the widespread transcription and cell-type specificity of lncRNAs in healthy human immune cells, which may be relevant to their altered function and development in the lung tumour microenvironment.

### Expression of immune lncRNAs can influence the anti-lung tumour response of NK cells

**Figure 4:**
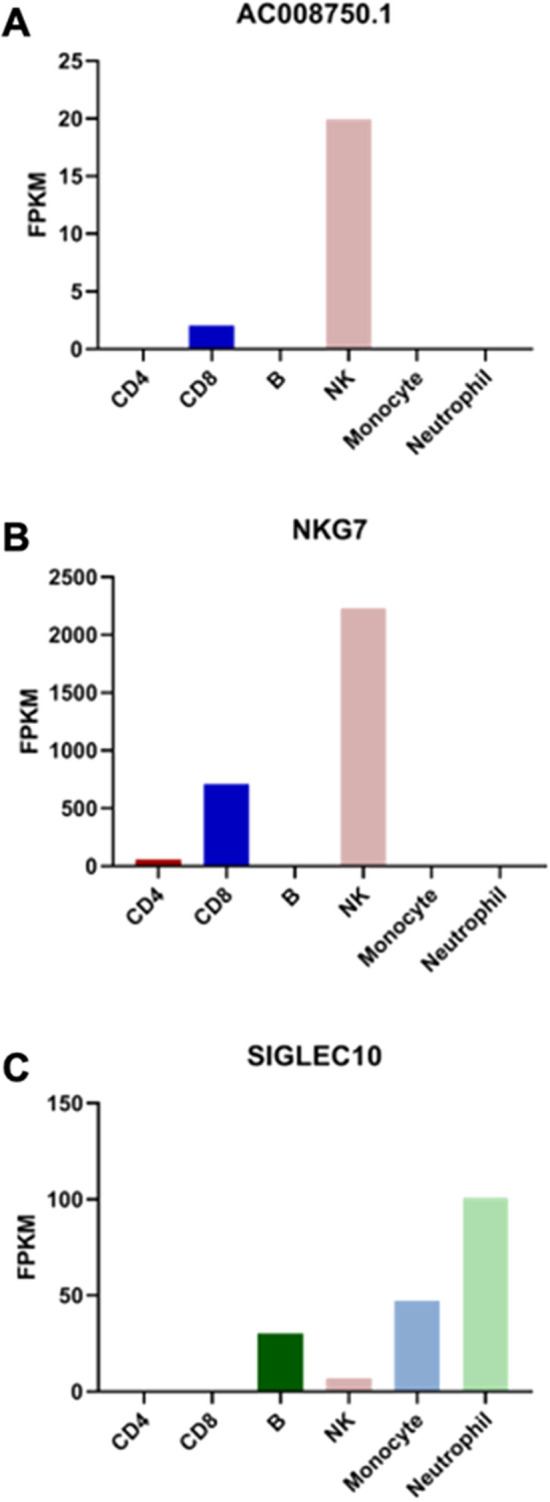
In silico expression of immune-lncRNAs may influence known immune-associated protein-coding genes. (**A**) Expression patterns of the lncRNA *AC008750.1*. (**B**) Expression of the protein-coding gene *NKG7* neighbouring *AC008750.1.* (**C**) Expression of overlapping protein-coding gene *SIGLEC10,* illustrating its discordant expression with *AC008750.1*.

In order to investigate the role of these tumour microenvironment-derived lncRNAs in anti-tumour immunity, we assessed their expression patterns between functionally-related immune cell types in the GSE60424 cohort. Through this analysis, we identified the 492 base pair *AC008750.1* non-coding transcript, located on chromosome 19q13.41 as annotated by Ensembl. We found that this transcript is exclusively expressed in healthy CD8 + T cells and NK cells, which share analogous cytotoxic function (Fig. 4A). Strikingly, this lncRNA is located approximately 20 kb upstream of *NKG7*, which codes for the protein NKG7/GMP-17. NKG7 is involved in the granulation response of both NK cells and cytotoxic T cells, and is a marker of cytotoxic effector function in CD8 + T cells^34–36^. *AC008750.1* displayed concordant expression with *NKG7*, with high expression in NK cells, intermediate expression in CD8 + T cells, and little to no expression in all other cell types (Fig. 4B). To rule out the possibility that expression is due to amplification of this entire genomic locus, we analyzed the expression of *SIGLEC10*, another immune-related gene that directly overlaps *AC008750.1* on the chromosome. *SIGLEC10* displays high expression in B cells, granulocytes, and monocytes, intermediate expression in NK cells, and low expression in both CD4 + and CD8 + T cells (Fig. 4C), indicating that concordant expression of *AC008750.1 *and *NKG7* cannot be attributed to global amplification of this genomic region. Consequently, these findings may be suggestive of a *cis*-regulatory relationship between *AC008750.1* and *NKG7*.

**Figure 5:**
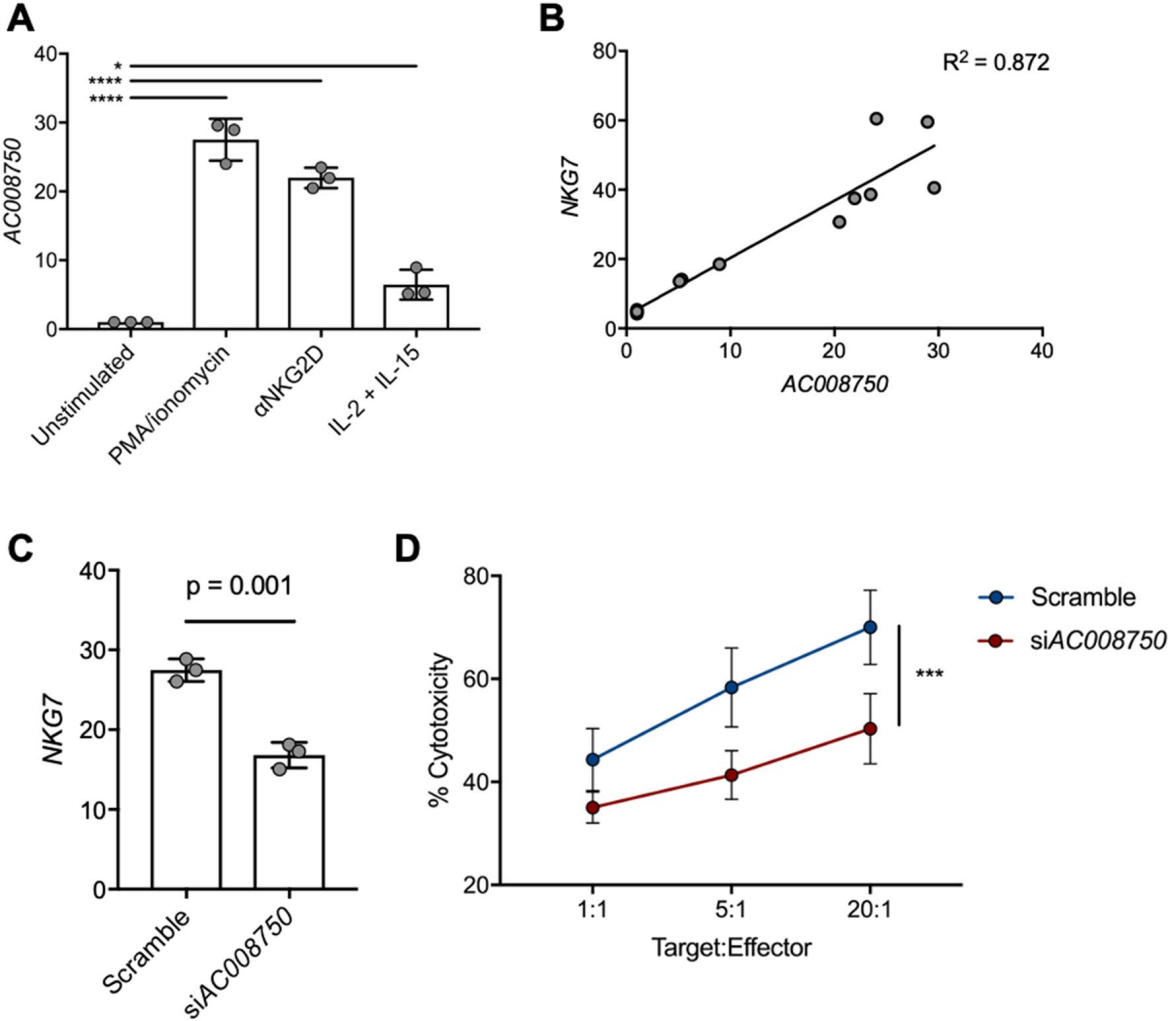
Expression of immune-related lncRNAs influences the anti-tumour immune response in vitro. (**A**) *AC008750.1* is upregulated in NK cells upon activation with various cytotoxic agents, as measured by qPCR. DeltaCT values are shown. (**B**) Expression of *AC008750.1* is significantly correlated with *NKG7* expression in NK cells. (**C**) *AC008750.1* knockdown decreases *NKG7* expression. (**D**) Knockdown of *AC008750.1* impairs in vitro killing of lung adenocarcinoma cell line (A549).

To further explore the basis for the concordant expression between *AC008750.1* and *NKG7*, we sought to experimentally validate the putative effect of this immune-related lncRNA on the anti-tumour response. Interestingly, in vitro stimulation of the NK cell line NK92 with PMA*/*ionomycin, anti-NKG2D, or culture with IL-2 and IL-15 was sufficient to induce expression of *AC008750.1,* as determined by qPCR (Fig. 5A). Upregulation of *AC008750.1* positively correlated with *NKG7* expression under these stimulatory conditions (Fig. 5B), in agreement with our in silico findings. We subsequently performed siRNA knockdown of *AC008750.1* in NK92 cells and found that upon PMA*/*ionomycin stimulation these cells were less able to upregulate *NKG7* compared with cells transfected with a non-targeting control siRNA (Fig. 5C). Finally, we co-cultured NK92 cells with the lung adenocarcinoma cell line A549 in order to assess the anti-lung tumour capacity of these cells. Across all target:effector ratios tested, *AC008750.1* knockdown cells displayed significantly lower cytotoxic capacity than those transfected with a non-targeting control (Fig. 5D). This provides functional evidence that decreased expression of *AC008750.1* is associated with impaired NK cell function, and suggests that expression of immune-derived lncRNAs may influence anti-lung tumour immunity, which should be further explored in future studies.

## Discussion

Here, we show that long non-coding RNA expression is widespread in healthy and tumour-infiltrating human immune cells. Though the mechanisms by which lncRNAs contribute to deregulated oncogenic gene regulation in tumour cells are well-studied, their role in infiltrating immune cells remains understudied. Recent studies have highlighted the prognostic role of immune cell-derived lncRNAs in the classification of immunogenic tumour subtypes as well as in stratifying patients for response to immunotherapy^25,37^. Our observations are consistent with these studies, and contribute to the increasingly recognized role of lncRNAs in lung tumour immunity.

Beyond their roles in tumour-infiltrating cells, we were able to show that lncRNA expression in healthy sorted immune cells was largely cell-type specific and recapitulated haematopoietic differentiation trajectories. Previous studies have demonstrated mechanistic roles for select lncRNAs in the development, maturation, and effector function of immune cells, and we provide here a comprehensive atlas of expressed lncRNAs in healthy human immune cells. Given the ability of lncRNA expression patterns to accurately cluster related immune cell types, we also suggest that lncRNAs can supplement existing deconvolution algorithms that infer immune cell abundance based on bulk RNAseq data^10,16,38,39^.

Our analysis of dysregulated lncRNA expression in tumours between microdissected and non-microdissected cohorts illustrates that lncRNAs detected in bulk lung tumour sequencing data may be indicative of the presence of infiltrating lymphocytes. Notably, lncRNA expression was found to be associated with the tumour cell purity of the sample; correlation of these genes with *CD45* and LUMP score suggested their immune cell origin. Given the biological importance attributed to lncRNAs that are observed to be deregulated in bulk tumour samples, we caution that components of the tumour microenvironment, including but not limited to infiltrating immune cells, likely confound lncRNA expression analysis of bulk tumour data as it does for protein-coding genes^40^. The advent of single-cell sequencing technologies and public availability of these data will aid in the accurate mapping of transcripts to their cell-of-origin. Nevertheless, our results in tandem with recent studies describing expression of lncRNAs in the lung tumour microenvironment emphasize that the contribution of cancer cell-extrinsic genes can confound tumour-sequencing efforts and must not be discounted^41^.

We observed an immune-related lncRNA to be associated with patient outcome in LUAD, which aligns with the poor outcome for patients lacking the presence of specific lymphocytes^42^. The specific expression of lncRNAs may provide the advantage of limited off-target effects for novel therapeutic agents targeting lncRNAs. Importantly for lncRNAs, the RNA transcript acts as the functional unit rather than as an intermediate – as is the case for protein-coding mRNAs – which may make them amenable to RNA-based inhibitors such as antisense oligonucleotides (ASOs), as in the case of ASO-induced silencing of the lncRNA *MALAT1* and the subsequent blocking of metastasis formation^43^. Thus, further analyses examining the therapeutic relevance of lncRNAs such as *AC008750.1* and *linc00861* expression in immune cells in bulk tumours using fluorescence in situ hybridization are required. Nonetheless, the lncRNAs described here may be revealed to have translational utility as prognostic markers of immune cell infiltration or response to immunotherapy.

Analyzing the specific expression patterns of lncRNAs can be used to infer the potential functional roles of these transcripts. For instance, we observed *MEG3* – a lncRNA with known function in the immune system in the regulation of the release of IL-1β – to be highly expressed in monocytes^44^. Further, as lncRNAs are known to influence gene expression *in cis*, it is similarly important to include an assessment of genomic location and neighbouring gene expression patterns when examining putative regulatory relationships for a given lncRNA. In our analysis, we observed the lncRNA transcript *AC008750.1*, a lncRNA with no ascribed functional roles, to be expressed in cytotoxic immune cells. When examining the genomic location of this lncRNA, we noticed *NKG7* to be transcribed from a neighbouring genomic locus. Interestingly, *NKG7* is an important immune-related gene, involved in the effector function of cytotoxic cells and the granulation response. In our observations, we found *NKG7* and *AC008750.1* to display concordant expression patterns, lending support to a possible regulatory relationship between these coding and non-coding transcripts. It is often the case that these observations are simply the result of the upregulation of an entire genomic locus, however, the gene overlapping *ACC08750.1*, *SIGLEC10* – also involved in the immune response – had a unique expression pattern not congruent with either *AC008750.1* or *NKG7*. Thus, the transcription of *ACC08750.1* is unlikely the result of passenger effects. Indeed, siRNA-mediated knockdown of *AC008750.1* was sufficient to reduce the cytotoxic capacity of NK cells in co-culture with lung tumour cells, which was associated with a deficiency in *NKG7* upregulation. *AC008750.1* is only one lncRNA transcript that we have demonstrated to have in vitro anti-tumour activity, however, these results set a precedent for the inquiry of many similar regulatory relationships in other functional domains, in light of the widespread mechanisms used by lncRNAs to exert their regulatory effects on protein-coding genes^12^. As these observations demonstrate the impact of lncRNA deregulation on the cytotoxic activity of anti-tumour cells, future studies may seek to explore impaired lncRNA expression and subsequent disruption of important gene-regulatory pathways as a means whereby tumours are able to evade the immune system.

Together, these results highlight the contribution of infiltrating immune cells to gene expression data from bulk samples. Immune-derived lncRNAs may be used to identify the presence of specific immune cell populations within tumour samples from bulk and single-cell RNA sequencing data, as well as provide novel explanations for observed deregulated expression patterns of genes deregulated in cancer. Thus, the expression patterns of these lncRNAs in healthy and malignant tissue provide a novel resource to better understand the mechanism of immune cell-specific gene regulation in cancer.

## Supplementary information

Supplementary file 1

Supplementary file 2

Supplementary file 3

Supplementary file 4

Supplementary file 5

Supplementary file 6

Supplementary file 7

Supplementary file 8

Supplementary file 9

Supplementary file 10

Supplementary file 11

## Data Availability

The data that support the findings of this study are available, but restrictions apply to the availability of these data, which were used under licence for the current study, and as such are not publicly available. Data are however available from the authors upon reasonable requests and with permission.

## Abbreviations

lncRNA: Long non-coding RNA
NK: Natural killer
NSCLC: Non-small cell lung cancer
IHC: Immunohistochemistry
LUAD: Lung adenocarcinoma
BCCRC: BC Cancer Research Centre
TCGA: The Cancer Genome Atlas
PMA: Phorbol 12-myristate 13-acetate
ANOVA: Analysis of variance
scRNAseq: Single cell RNA sequencing
LUMP: Leukocytes unmethylation for purity
qPCR: Quantitative polymerase chain reaction
ASO: Antisense oligonucleotide
