# Supplementary figures and images for: Assessment of long non-coding RNA expression reveals novel mediators of the lung tumour immune response
Source: Sci Rep. 2020 Oct 9;10:16945. doi: 10.1038/s41598-020-73787-6 (PMC7547676; doi:10.1038/s41598-020-73787-6)

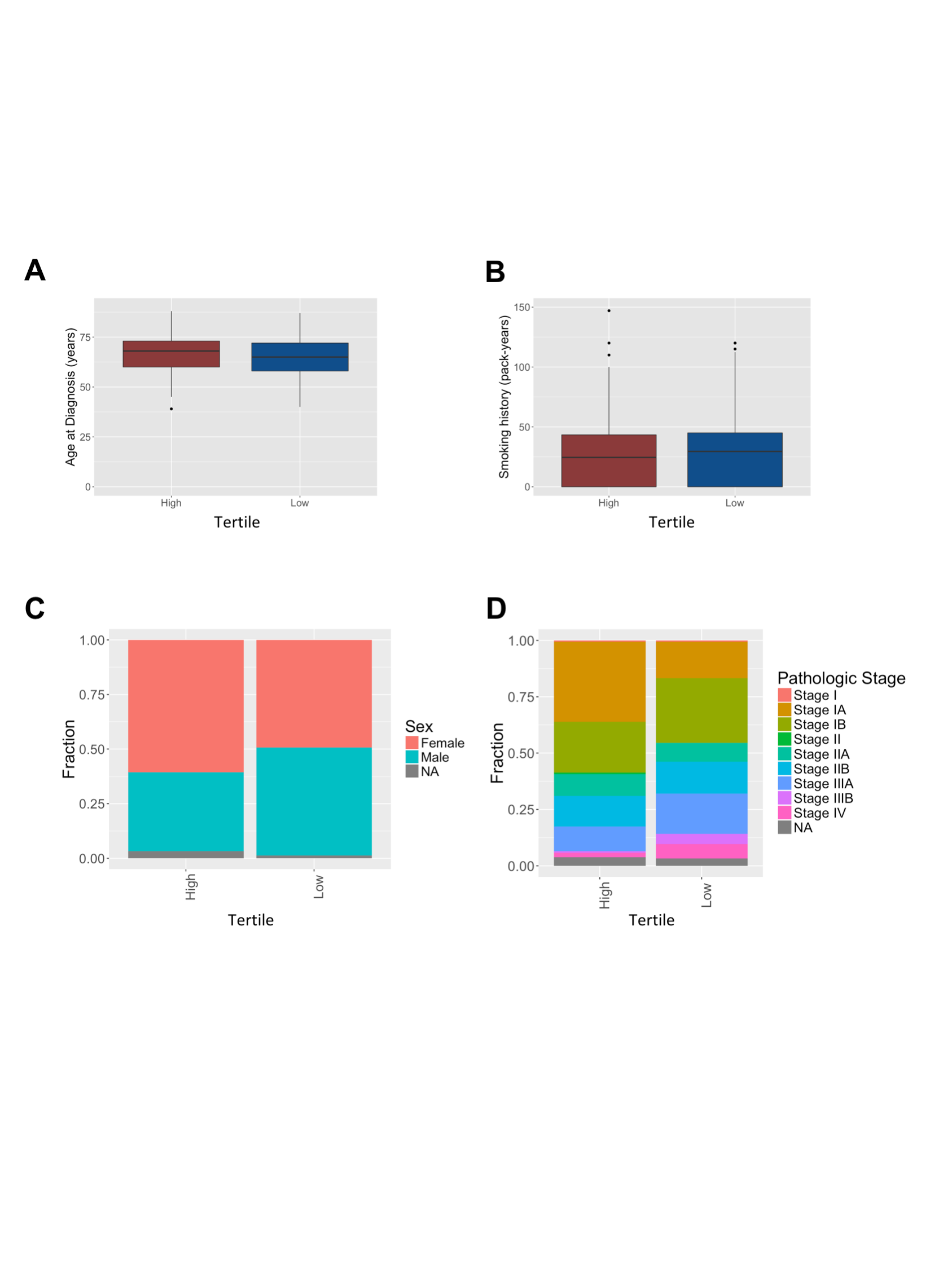

Supplement: Supplementary file 1 [file 41598_2020_73787_MOESM1_ESM.tiff]
