# Supplementary material for: Assessment of long non-coding RNA expression reveals novel mediators of the lung tumour immune response
Source: Sci Rep. 2020 Oct 9;10:16945. doi: 10.1038/s41598-020-73787-6 (PMC7547676; doi:10.1038/s41598-020-73787-6)
Supplement: Supplementary file 11 [file 41598_2020_73787_MOESM11_ESM.docx]

**Fig S1. Analysis of clinical covariates with respect to *linc00861* expression.** A) Age at diagnosis in *linc00861*-high and –low patients, ranked by tertiles. B) Smoking history in *linc00861*-high and –low patients, ranked by tertiles.  C) Patient sex in *linc00861*-high and –low patients, ranked by tertiles.  D) Pathologic stage in *linc00861*-high and –low patients, ranked by tertiles.
